# HBV-miR-3 induces hepatic cholesterol accumulation by targeting *ABCA1*: Evidence for potential benefits of statin usage

**DOI:** 10.1016/j.jlr.2025.100866

**Published:** 2025-07-21

**Authors:** Shruti Chowdhari, Auroni Deep, Belal Ahmad, Jasmine Samal, Ekta Gupta, Perumal Vivekanandan

**Affiliations:** 1 Kusuma School of Biological Sciences, Indian Institute of Technology Delhi, New Delhi, India; 2 School of Biological Sciences, Doon University, Uttarakhand, India; 3 Institute of Liver and Biliary Sciences (ILBS) Repository, New Delhi, India

**Keywords:** hepatitis B virus, HBV-miR-3, *ABCA1*, hepatic steatosis, HCC, cholesterol

## Abstract

The cellular targets of hepatitis B virus (HBV)-encoded miRNAs remain poorly understood. The evolutionary conservation of HBV-miR-3 across HBV genotypes suggests its potential functional importance. Transcriptome profiling of HBV-miR-3 expressing hepatocytes demonstrates differential expression of several genes associated with lipid metabolic processes. The cholesterol efflux regulator gene *ABCA1* was found to be downregulated in our microarray data and GEO datasets from HBV-infected liver. We validated *ABCA1* as a bona fide target of HBV-miR-3. HBV-miR-3-mediated suppression of *ABCA1* led to increased cholesterol and lipid droplet accumulation in addition to increased proliferation and colony formation in hepatocyte cell lines. Interestingly, widely prescribed cholesterol-lowering drugs (simvastatin, atorvastatin, and fluvastatin) could inhibit the pro-oncogenic effects of HBV-miR-3. HBV-miR-3 expression was detectable in all liver biopsies (n = 20) from patients with chronic HBV (CHBV). Patients with high intrahepatic HBV loads had higher levels of HBV-miR-3, suggesting that the virus-encoded miRNA levels correlate with virus replication. Patients with high HBV-miR-3 expression had significantly lower *ABCA1* transcript levels in the liver. Hepatic steatosis was more frequently observed in biopsies of patients with high intrahepatic HBV-miR-3 levels compared to those with low HBV-miR-3 levels (71% vs. 53%), although this was not statistically significant. Taken together, our findings support the notion that HBV-miR-3-mediated suppression of *ABCA1* contributes to dysregulation of lipid metabolism in CHBV infection. In sum, HBV-miR-3 may represent the “missing link” between CHBV and altered lipid metabolism in hepatocytes. Statin-mediated inhibition of HBV-miR-3-induced intrahepatic lipid accumulation and cell proliferation has potential clinical utility and merits further investigation.

## Graphical Abstract

**Figure:**
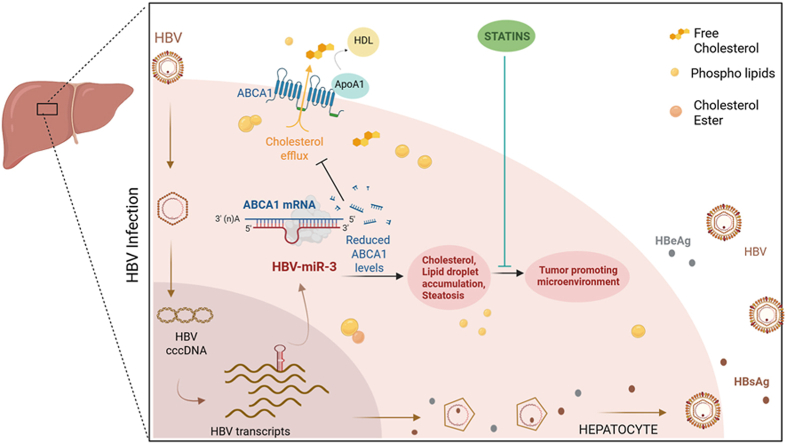

Hepatocellular carcinoma (HCC) is the third-leading cause of cancer-related deaths worldwide. Chronic HBV (CHBV) infection is a major risk factor for development of HCC. CHBV infection may lead to progressive hepatic steatosis, fibrosis and advanced cirrhosis culminating in HCC. Despite the availability of an efficacious vaccine, there are nearly 300 million individuals with CHBV infection (1, 2). Therefore, it is essential to understand the mechanisms underlining HBV-host interactions; the majority of published research focusses on HBV-encoded proteins. Although HBV-encoded miRNAs have been reported (3, 4), their role, if any, in the etiopathogenesis of HBV-related HCC remains poorly understood.

HBV is a non-cytopathic (5), hepatotropic virus belonging to the *Hepadnaviridae* family. The partially double-stranded circular DNA genome of HBV encodes four overlapping open reading frames, which encode seven proteins (5, 6). Previous research has demonstrated a role for HBV-encoded proteins (e.g. HBsAg, HBeAg, and HBx), HBV DNA secondary structures as well as cellular factors in cellular transforming events that involve immune evasion, ubiquitination, autophagy, and epigenetic modifications as well as regulation of virus gene expression, hepatic inflammation and fibrosis leading to hepatocarcinogenesis (7, 8, 9, 10, 11, 12). MicroRNAs are an important class of endogenous, small, non-coding functional RNA molecules transcribed from within intergenic regions, introns, or exons of protein-coding genes and are processed via canonical (13) or non-canonical (14, 15) pathways. Mature miRNAs (17-25 nt) typically interact with target mRNA 3′UTRs with a partial or complete seed (2–8 nt of miRNA sequence from the 5′ end) complementarity and, in association with RNA-induced silencing complex (RISC), induce mRNA decay or translational repression resulting in downregulation of the target gene expression. MicroRNAs regulate a plethora of biological processes (16). Next-generation sequencing has been used to identify HBV-encoded miRNAs in HBV-infected liver tissues (3, 4). HBV-miR-3, a HBV-encoded miRNA, is derived from the 5′ stem of the hairpin secondary structure formed in the 3.5kb, 2.4kb, and 2.1kb HBV transcripts within the hepatitis B surface antigen (HBsAg) coding ORF during HBV replication. HBV-miR-3 has been shown to control HBV replication by repressing its own transcripts (i.e., HBV 3.5kb transcript, pgRNA, and replication intermediates) (3) and, therefore speculated to contribute to chronic HBV infection. Few computationally predicted target genes *SOCS5* (17), *PPM1A* (18), *PTEN* (19) and *FIH-1* (20) were shown to be regulated by HBV-miR-3 in host cells. A recent study shows the prognostic significance of HBV-miR-3 as a predictor of HBsAg seroconversion in patients undergoing PegIFN-α treatment (21). In this study, we (a) identify ATP-binding cassette subfamily A member 1 (*ABCA1*) as a bonafide target of HBV-miR-3, (b) demonstrate how HBV-miR-3 leads to the dysregulation of lipid metabolism in hepatocytes by facilitating intracellular cholesterol accumulation, (c) identify the potential role of the HBV-miR-3-ABCA1 axis in hepatocarcinogenesis, and (d) show that the HBV-miR-3 mediated oncogenic effects can be inhibited by statins which are widely prescribed cholesterol lowering drugs.

## Materials and Methods

### Conservation analysis of HBV-miR-3 seed region

All available HBV surface gene sequences from HBV-genotypes A to H (total: 26,278; genotype A: 3,498; genotype B: 6,166; genotype C: 10,079; genotype D: 4,829; genotype E: 897; genotype F: 562; genotype G: 157; genotype H: 90) were retrieved from the HBV database (https://hbvdb.ibcp.fr/HBVdb/). The nucleotide stretches encoding the HBV-miR-3 seed sequence were extracted, and a multiple sequence alignment was performed using BioEdit v7.2.5 tool.

### Cell culture and transfections

Human liver cancer cell lines: Huh7 (hepatocellular carcinoma) and HepG2 (hepatoblastoma) were procured from National Center for Cell Science (NCCS) Pune, India and maintained in DMEM (Invitrogen) containing 10% (v/v) fetal calf serum, 100 units ml^-1^ penicillin, 100 μg ml^-1^ streptomycin, 0.25 μg ml^−1^amphotericin (Invitrogen) at 37°C in a humidified atmosphere at 5% CO_2._ Transfections were carried out using Lipofectamine 2,000 (Invitrogen) according to the manufacturer’s protocol. The statins: simvastatin, atorvastatin (TCI Co., LTD), and fluvastatin (MCE) were dissolved in dimethyl sulfoxide (DMSO; Sigma-Aldrich) and diluted to working concentrations in the complete cell culture media.

### Plasmid constructs and luciferase reporter assays

For overexpression of HBV-miR-3 in hepatocyte cell lines, the genomic region within the HBV S gene encoding the HBV-miR-3 hairpin (precursor miRNA) was amplified from HBV replication-competent plasmid (HBV1.3x) and cloned between the restriction sites *HindIII* and *XhoI,* downstream of the CMV promoter in the pcDNA3.1+ mammalian expression vector; we refer to this construct as pHBV-miR-3. The empty vector (pcDNA3.1+) was used as a vector control for all experiments. The 3′-untranslated region (UTR) of the human *ABCA1* gene harboring the predicted HBV-miR-3 response element was cloned between restriction sites *AsiSI* and *XhoI* downstream of the *Renilla* luciferase reporter gene in the psiCHECK-2 dual luciferase vector and designated as pWT-ABCA1 UTR. The 8-nt HBV-miR-3 response element was deleted from the pWT-ABCA1-UTR with the help of QuikChange site-directed Mutagenesis Kit (Agilent Technologies), and the resultant plasmid is referred to as pMut-ABCA1-UTR. All plasmids were sequenced for confirmation. For luciferase reporter assays, cells were co-transfected with 300 ng of either pWT-ABCA1 UTR or pMut-ABCA1 UTR along with 300 ng of pHBV-miR-3 or the vector control. *Renilla* and *Firefly* luciferase activities were measured after 48 h of transfection by using the Dual-Luciferase Reporter Assay System (Promega). *Renilla* luciferase activity was normalized to that of *firefly* luciferase, and data were plotted as relative luciferase units (RLU).

### Transcriptome profiling

Total RNA was extracted from Huh7 cells expressing HBV-miR-3 as well as control cells (cells transfected with the empty vector) using Trizol reagent (Invitrogen). Two-hundred and fifty ng of total RNA was used for cDNA synthesis using T7 oligo(dT) primers followed by synthesis of second strand cDNA from which biotinylated complimentary RNA (cRNA) was prepared using the GeneChip 3′ IVT Plus Reagent Kit (Affymetrix). The biotinylated cRNA was subjected to fragmentation followed by hybridization to the Gene Chip Prime View™ Human Gene Expression Array cartridges for 16h at 45^°^C. The array cartridges were stained and washed using the Affymtrix Gene Chip Fluidics Station 450 (Affymetrix). Subsequently, scanning of array cartridges was done using Affymetrix Gene Chip Scanner 3,000 7G (Affymetrix) to obtain fluorescence intensity data. The fluorescence intensity data in CEL format was converted to.CHP file using the Applied Biosystems™ Transcriptome Analysis Console (TAC) Software (v4.0.2) wherein the background correction, quantile normalization and summarization was performed using Robust Multichip Analysis (RMA) and the differential gene expression was analysed using Limma R package (version 3.46.0). The differentially expressed genes (DEGs) which crossed the threshold of false discovery rate (FDR) < 5%, fold change (FC) ± 1.25 and *P value* ≤ 0.05 were considered statistically significant. GeneCodis4 tool (https://genecodis.genyo.es/) was used for gene ontology enrichment analysis. The tool employs hypergeometric test for calculating *P-*values and false-discovery rate (FDR) for *P-*value correction.

### In silico prediction of host genes with potential HBV-miR-3 binding sites

Host genes with potential HBV-miR-3 response elements (binding sites) were computationally predicted using the TargetScan Custom v5.2 algorithm (https://www.targetscan.org/vert_50/) (22). The minimum free energy (MFE) for the potential miRNA:mRNA pairs was computed using the RNAhybrid tool (23).

### Quantitative reverse transcription polymerase chain reaction (qRT-PCR)

Total RNA was extracted using TRIzol reagent (Invitrogen), treated with DNase 1 (NEB) and then reverse transcribed using Super Reverse Transcriptase MuLV Kit (BioBharati Life Science Pvt. Ltd) as per the manufacturer’s instructions. Quantitative reverse transcription-polymerase chain reaction (qRT-PCR) was carried out with iTaq Universal SYBR Green Supermix (BioRad Laboratories) in triplicate in a C1000 Thermal cycler (BioRad laboratories). All data were normalized by using 18s rRNA as an internal control. The details of primers used are listed in supplemental Table S1.

### Immunocytochemistry

Huh7 and HepG2 cells were transfected with pHBV-miR-3 or pcDNA3.1+. After 48 h, the cells were fixed with 4% PFA for 10 min, permeabilized with 0.1% Triton X-100 for 10 min, followed by blocking in 5% BSA for 1h. For immunofluorescence staining, ABCA1 Antibody (1276B) [NBP2-54792, Novus Biologicals] was used at a 1:100 dilution overnight, and Alexa Fluor 555 goat anti-rabbit IgG [A-11008, Thermo Fisher Scientific] at a 1:800 dilution for 2 h was used as a secondary antibody. All cells were counterstained using 4′,6-diamidino-2-phenylindole (DAPI) (ThermoFisher Scientific), for 10 min at room temperature to visualize cell nuclei and mounted with Antifade mounting medium (ThermoFisher Scientific). Images were captured using an inverted microscope (Radical Scientific, India) with a minimum of 7 fields of view per treatment and quantified as a percentage of total DAPI-positive nuclei using ImageJ software.

### Total cholesterol estimation

The treated cells were harvested, washed with PBS and divided into two aliquots. One aliquot was used for protein determination by the bicinchoninic acid protein assay (ThermoFisher Scientific). The second aliquot was subjected to lipid extraction as described earlier (24) followed by estimation of total cellular cholesterol content using the Amplex red cholesterol fluorometric assay kit (ThermoFisher Scientific). Briefly, the lipid extracts were dried in a speed-vac, re-suspended in 50 μl of reaction buffer after which an equal volume of Amplex red working solution (300 μM Amplex Red, 0.2 U/ml cholesterol esterase, 2U/ml cholesterol oxidase and 2U/ml horse radish peroxidase) was added. The samples were incubated for 30 min at 37°C in dark. The fluorescence was measured at 590 nm in accordance with the manufacturer’s protocol. The intracellular cholesterol values were normalized by protein content.

### Lipid droplet analysis

The lipid droplet accumulation in cells was analysed using lipophilic dyes BODIPY 493/503 and Oil Red O. For BODIPY 493/503 staining, cells (after 48h of transfection with pcDNA3.1+ or pHBV-miR-3) were fixed with 2% PFA for 30 min, washed with PBS and then simultaneously treated with BODIPY 493/503 (10 μM) and DAPI (1 μg/ml) for 15 min. Cells were washed, then mounted with Antifade mounting medium (ThermoFisher Scientific). Images were captured using an inverted epifluorescence microscope (RTC-7 NXF; Radical Scientific) at 40X magnification with a 6 fields of view per sample. Oil Red O (ORO as 0.5% w/v stock solution) was prepared in isopropanol with gentle heating and diluted in distilled water in the ratio 3:2, then filtered before use. Two days post-transfection, the cells were washed with PBS and fixed in 2% paraformaldehyde for 10 min at room temperature. The cells were rinsed with 60% isopropanol (for dehydration) and stained with ORO solution for 45 min, followed by another rinse with 60% isopropanol to remove excess stain. The cells were washed with distilled water three times. The experiment was set up in duplicate. One set of cells was used for quantifying ORO-incorporated intracellular lipids wherein the ORO in stained cells was eluted by addition of absolute Isopropanol (500 μl per each well of 6 well plate, on rocker for 5 min), the ORO containing extracts were then transferred to 96 well plate and the absorbance was measured at 518 nm as previously described (25). The second set of cells was stained with ORO, counterstained with Myer's hematoxylin, mounted with Aquatex, and then visualized at 40X magnification using a brightfield inverted microscope (DEBRO DIM-150).

### Cell proliferation assay

The viability of hepatocytes expressing HBV-miR-3 compared to those transfected with vector controls was determined by MTT [3-(4, 5-dimethylthiazol)-2, 5-diphenyltetrazolium bromide] assay at 48 and 72 h. Both Huh7 and HepG2 cell lines were transfected with pHBV-miR3 or pcDNA3.1+, and 48 or 72h later, treated with MTT for 45 min in the dark. The media was discarded and the formazan crystals were dissolved in 100 μl DMSO. The optical density (OD) was measured at 570 nm using a plate reader. Cell percentage viability of the cells was calculated using the formula: %cell viability = (mean OD in test wells/mean OD in control wells) X 100.

### Colony-forming assay

For colony forming assay, the Huh7 and HepG2 cell lines were transfected with pHBV-miR-3 or pcDNA3.1+, 24 h later the cells were trypsinized and seeded into six-well plates at a density of 5,000 cells/well and were maintained in the CO_2_ incubator at 37°C for 10 days. Subsequently, the cells were fixed with ethanol (95%) for 30 min followed by staining with crystal violet (0.25% in 2% ethenol) for 15 min. The cells were washed with distilled water and imaged using the Gel Doc XR + System (Bio-Rad Laboratories). For quantification, the Crystal violet stain was eluted in 30% Glacial acetic acid and the absorbance was measured at 570 nm using microplate reader (ThermoFisher Scientific).

### Cell migration assay

Huh7 and HepG2 cell lines transfected with pHBV-miR-3 or pcDNA3.1+ were grown till full confluence in 6-well culture plates and a scratch was made using a sterilized 10 μL pipette tip, followed by the addition of fresh media. The scratch area was photographed at 10X magnification using the inverted optical microscope (Invitrogen). The scratch area was measured with the help of the ImageJ software.

### Liver biopsy specimens of patients with CHBV infection

Fresh frozen liver biopsies from patients with CHBV infection (n = 20), their respective serum lipid profiles, and histopathological findings were retrieved from the Institute of Liver and Biliary Sciences (ILBS) repository, Delhi, India, after approval from the Institute Ethics Review Committee (Approval number: IEC/2023/98/MA09). The study was performed in agreement with the principles set by the *Declaration of Helsinki.* The patient cohort included 19 males and 1 female with a median age of 51 (range: 32–64 years). All the patients were positive for HBsAg or HBV-core and negative for HCV, HIV, and HDV. The liver biopsies were homogenised using the bead beater and divided into two parts, one part for DNA extraction and the other for RNA extraction. Total DNA was extracted using the QIAamp DNA mini kit (QIAGEN GmbH). Total RNA was extracted using the TRIzol reagent (Invitrogen) according to the manufacturer’s instructions. The HBV-DNA amount was normalized with human beta-globin (hβ-globin), and the HBV-miR-3 copy number was normalized with 18s rRNA copy number in every biopsy sample. The copy numbers were determined using standard curve analysis (standard curves were analysed using serial dilutions of cloned plasmid DNA ranging from 10^5^ to 10^1^ copies/reaction in triplicate. Linearity in standard curves was observed between dilutions 10^5^ to 10^2^, Supplementary information (supplemental Fig. S6). No signal was observed in the no-template controls). CHBV patients with intrahepatic HBV-miR-3 levels ≥ 10^2^ copies/reaction (i.e., within the linear range) were classified as those with “high HBV-miR-3”. Patients with intrahepatic HBV-miR-3 levels of < 10^2^ copies/reaction were classified as those with “low HBV-miR-3”. All qRT-PCR primers are listed in supplemental Table S1.

### Statistical analysis

The Spearman’s rank correlation was used to determine a significant relationship between the intrahepatic HBV-miR-3 levels and hepatic HBV DNA levels (viral load). The mean values were compared using Student's *t* test. Origin 9.7.5 (Origin Lab Corp) was used for violin plots. Mann–Whitney *U* test was performed to compare the medians in the violin plots. The chi-square test was performed to compare the steatosis incidence between the groups. Values of *P* ≤ 0.05 were considered statistically significant.

## Results

### Conservation of HBV-miR-3 seed within and across HBV genotypes

**Fig. 1:**
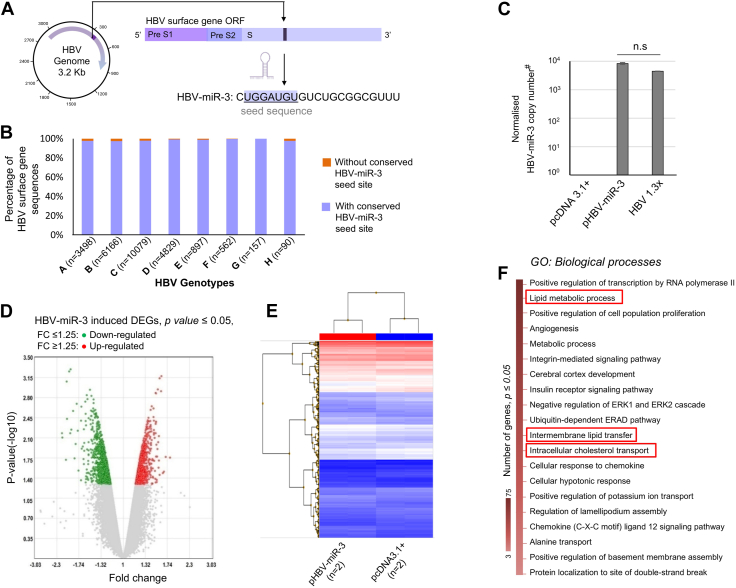
Functional importance of HBV-miR-3 in HBV pathogenesis. A: Graphical representation of the HBV genomic region (HBV Surface gene) encoding HBV-miR-3. *B*, conservation analysis of HBV-miR-3 seed site across and within HBV genotypes. Each bar graph represents the percentage (%) of sequences with and without the conserved seed site for HBV-miR-3 (purple and orange bars respectively). C: qRT PCR detection and quantification of mature HBV-miR-3 copies in Huh7 cell line transfected with HBV-miR-3 overexpression construct (pHBV-miR-3), pcDNA3.1+ (Vehicle control), HBV 1.3x (positive control). The bar graphs represent mean (±SD) of three independent experiments. (# HBV-miR-3 normalization: HBV-miR-3/18srRNA/100 ng RNA, ∗*P* ≤ 0.05.) D: Volcano plot representing the DEGs between pHBV-miR-3 and pcDNA3.1+ transfected cells. The data points represented by green (down-regulated) and red (up-regulated) dots represent DEGs which crossed the filtering threshold of fold change (FC) ± 1.25 and *P* ≤ 0.05. E: Heat Map representing the hierarchical clustering of DEGs between cells overexpressing HBV-miR-3 and controls (F) Gene Ontology: Biological processes enriched by DEGs. n.s.: non-significant.

The miRNA seed (2-8 nt sequence from the 5′ end of the mature miRNA) plays an important role in specifying the complementarity-directed miRNA: mRNA target associations (16). Alignment of all available HBV surface gene sequences encompassing the coding region for HBV-miR-3 seed (Fig. 1A), extracted from HBV database (n = 26,278) showed that the HBV-miR-3 seed site is present in >97.7% of sequences from all HBV genotypes, as shown in Figure 1B. The conservation of HBV-miR-3 seed site, across HBV genotypes suggests a potential functional role for this virus-encoded miRNA.

### HBV-miR-3-induced changes in the hepatocyte transcriptome

To understand the changes in hepatocyte gene expression in response to HBV-miR-3, Huh7 cells transfected with HBV-miR-3 overexpression construct (pHBV-miR-3) or empty vector (pcDNA3.1+) were subjected to genome-wide transcriptome profiling. First, the expression level of mature HBV-miR-3 was confirmed in Huh7 cells transfected with pHBV-miR-3, HBV1.3x (positive control), and pcDNA3.1+ (empty vector; vehicle control) using stem-loop qRT-PCR. Mature HBV-miR-3 was detected in pHBV-miR-3 and HBV 1.3x-transfected cells but was not detected in cells transfected with the empty vector (Fig. 1C).

Our microarray data demonstrated differential expression of 768 genes in HBV-miR-3 expressing cells compared to controls (with fold change ± 1.25, *P* ≤ 0.05), including 158 upregulated genes and 610 downregulated genes (Fig. 1D). Hierarchical clustering of the HBV-miR-3-mediated differentially expressed transcripts is shown in Figure 1E.

Functional enrichment analysis of differentially expressed genes (DEGs) using the GeneCodis4 web tool indicates an overrepresentation of genes related to various biological processes including lipid metabolic processes (58 genes, *P* value = 1.79E-04), intermembrane lipid transfer (9 genes, *P* value = 2.94E-04) and intracellular cholesterol transport (5 genes, *P* value = 1.57E-04), (Fig. 1F and supplemental Table S2). The list of genes in these GO: Biological processes, along with the fold change in the array data, are provided in supplemental Table S2.1.

**Fig. 2:**
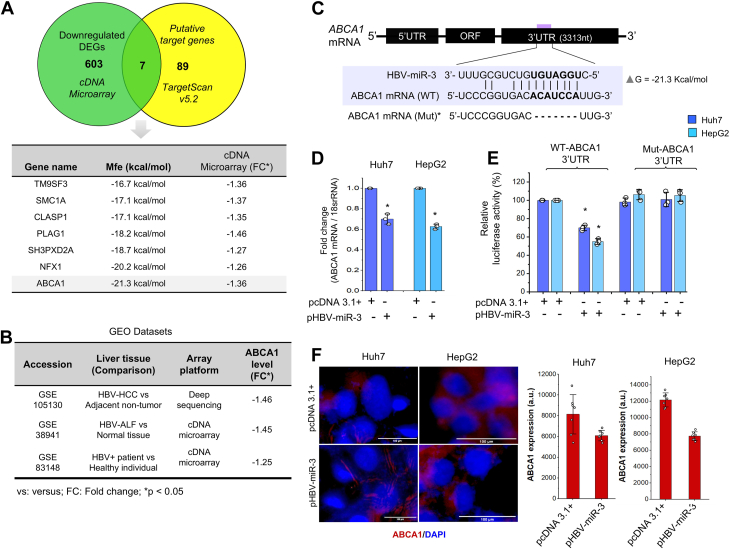
HBV-miR-3 downregulates h*ABCA1* expression. A: Upper panel: Venn diagram showing common genes between two data sets (a) downregulated DEGs (microarray data) and (b) computationally predicted host genes with putative HBV-miR-3 binding sites. Lower panel: Computed Mfe of binding between the potential miRNA-mRNA pairs and their respective expression level of the gene observed in the microarray data. B: Data extracted from published GEO Datasets: *ABCA1* transcript levels in HBV infected liver biopsies compared with normal liver. C: Schematic representation of predicted HBV-miR-3 binding site in *ABCA1* mRNA. D: qRT PCR analysis of *ABCA1* mRNA level in cells (Huh7, HepG2) transfected with pHBV-miR-3 compared to pcDNA3.1+ transfected cells. We used 18s rRNA was used as internal control. E: Analysis of luciferase reporter activity of ABCA1-3′UTR reporter constructs having either wildtype or deleted HBV-miR-3 binding sites in the presence or absence of HBV-miR-3 in both (Huh7, HepG2) cell lines. Firefly luciferase counts were normalized with *Renilla* luciferase counts. The bar graphs represent mean (±SD) of three independent experiments. F: Left panel: Immunostaining for ABCA1 protein in cells (Huh7, HepG2) transfected with pHBV-miR-3 compared to pcDNA3.1+ transfected cells. The cells were counterstained with nuclear stain DAPI. Right Panel: Fluorescence intensity was quantified in each micrograph and expressed as the mean ± standard errors (n = 7) in arbitrary units (a.u). Scale bar: 100 μM. ∗*P* ≤ 0.05.

Simultaneously, we also identified host genes harboring potential HBV-miR-3 binding sites using TargetScan Custom v5.2 and RNA-hybrid2.2 algorithms. The *in-silico* analyses revealed a total of 96 host genes with at least one 7-8mer HBV-miR-3 seed complementary site with the minimum free energy (Mfe) of the predicted miRNA: mRNA interaction ranging from −12.6 to −24.6 kcal/mol. We compared the list of host genes downregulated in the microarray (n = 610) with the list of host genes containing computationally predicted HBV-miR-3 binding site (n = 96) (Venn diagram, Figure 2A, -upper panel). We found seven genes that were present in both the lists (i.e., genes downregulated in the microarray dataset harbouring a computationally predicted HBV-miR-3 binding site) as listed in Figure 2A (lower panel). Of these genes, the cellular cholesterol exporter ATP binding cassette subfamily A member 1 (*ABCA1*) was found to have the lowest Mfe (−21.3 kcal/mol; Fig. 2A: lower panel).

### *ABCA1* is downregulated in HBV-infected livers

Interestingly, *ABCA1* was grouped under all the three GO: Biological process annotations (lipid metabolic process, intracellular cholesterol transport and intermembrane lipid transfer), indicating the possible modulation of aforementioned pathways by HBV-miR-3 via regulation of *ABCA1*. Hepatocytes play a central role in maintaining cholesterol and lipid homeostasis. *ABCA1*, an integral cell-membrane protein functions at the basolateral surface of polarized hepatocytes to facilitate the transfer of cellular excess free cholesterol and phospholipids across the plasma membrane. *ABCA1* is known to play a pivotal role in maintenance of intrahepatic cholesterol as well as plasma HDL level (26). To assess the clinical relevance of *ABCA1* expression levels in the context of HBV infection, we further extracted and analyzed the transcriptome profiles of HBV positive patients’ liver biopsies deposited on GEO (https://www.ncbi.nlm.nih.gov/geo/). The datasets GSE105130 (27), GSE38941 (28) and GSE83148 (29) comprised of transcriptome profiles of paired HBV associated liver tumor versus adjacent non-tumor (n = 24), HBV-associated acute liver failure (n = 4) versus liver donors (n = 10) and liver tissues from patients with positive HBsAg or serum HBV-DNA (n = 122) versus healthy samples (n = 6), respectively. We found *ABCA1* among the differentially downregulated genes in HBV infected liver tissues in all the three datasets. (Fig. 2B, supplemental Fig. S1). None of the other 6 genes (TM9SF3, SMC1A, CLASP1, PLAG1, SH3PXD2A, NFX1; Fig. 2A) showed consistent down-regulation in both HepG2 and Huh7 cells (supplemental Fig. S2). Hence, we sought to understand the role of HBV-miR-3-mediated regulation of ABCA1.

### Validation of *ABCA1* as a bona fide target of HBV-miR-3

We examined if *ABCA1* expression levels are modulated by HBV-miR-3 in hepatoma cells expressing HBV-miR-3. Figure 2C represents the predicted HBV-miR-3 response element in the 3′UTR of *ABCA1* mRNA. Our qRT PCR and immunostaining data confirmed that expression of HBV-miR-3 led to a significant decrease in *ABCA1* mRNA as well as protein levels respectively in both Huh7 and HepG2 cells compared to the controls (Fig. 2D, F; *P* ≤ 0.05). These results are concordant with our transcriptomics data and the transcriptomic data from HBV infected livers available in public databases. We used a luciferase reporter assay to confirm if *ABCA1* is a bonafide target of HBV-miR-3. HepG2 and Huh7 cells were co-transfected with *ABCA1-*3′UTR luciferase reporter constructs (i.e., pWT-ABCA1 UTR or pMut-ABCA1-UTR; Fig. 2C) and pHBV-miR-3. Expression of HBV-miR-3 significantly reduced the luciferase activity of pWT-ABCA1-3′UTR in both Huh7 and HepG2 cells (Fig. 2E; *P* ≤ 0.05). HBV-miR-3 did not have a significant effect on luciferase activity of pMut-ABCA1-3′UTR (Fig. 2E). These results confirm HBV-miR-3 bindis to *ABCA1* 3′UTR in hepatoma cell lines and *ABCA1* is a bona fide target of HBV-miR-3.

### HBV-miR-3 increases cellular cholesterol and lipid droplet (LD) accumulation

**Fig. 3:**
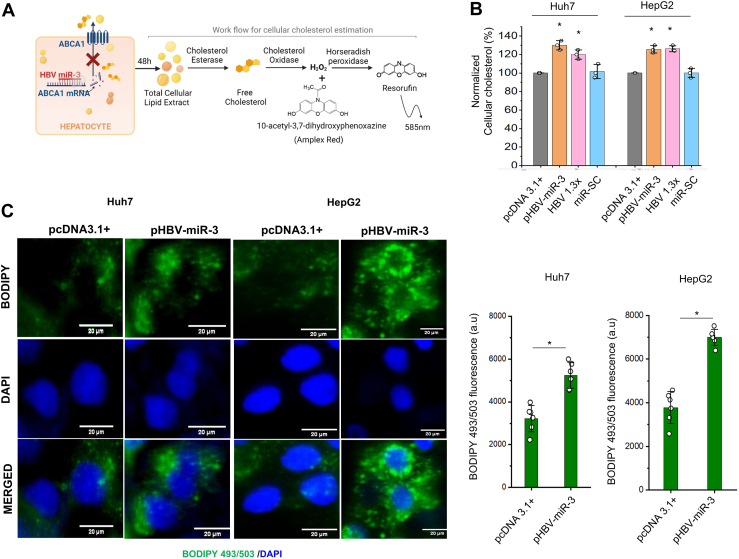
HBV-miR-3 expression leads to hepatocyte cholesterol build-up. A: Experimental design of the fluorometric cholesterol estimation assay performed for evaluating the expected increase in intracellular cholesterol post HBV-miR-3 expression in hepatocytes. B: Cellular cholesterol levels in cells (Huh7, HepG2) 48h after transfection with pHBV-miR-3, pcDNA3.1+, HBV 1.3x and microRNA-Scramble control (miR-SC). The cholesterol levels were normalised to respective total protein content. Bar graphs represent mean (±SD) of three independent experiments. C: Left panel: Hepatic lipid droplet accumulation visualised by BODIPY 493/503 staining in cells (Huh7, HepG2) 48 h post transfection with pHBV-miR-3 and pcDNA3.1+. The cells were counterstained with nuclear stain DAPI. Scale bar: 20 μM. Right panel: Quantitative analysis of BODIPY 493/503 fluorescence intensity was quantified in each micrograph and expressed as the mean ± standard errors (n = 6) in arbitrary units (a.u). ∗*P* ≤ 0.05.

Since *ABCA1* plays an important role in cholesterol trafficking across plasma membrane, we next examined if HBV-miR-3-mediated downregulation of *ABCA1* impacts the cellular cholesterol content. Total cholesterol levels in Huh7 and HepG2 cells transfected with pHBV-miR-3 were significantly higher (>30%) in both HepG2 and Huh7 cells as compared to the controls (pcDNA3.1+, microRNA-scramble control (miR-SC)). Notably, the cholesterol levels in HBV-miR-3 expressing cells is comparable to cholesterol levels in HBV expressing cells (cells transfected with HBV 1.3x). There is no significant difference in cholesterol levels between cells transfected with pcDNA3.1+ or miR-SC. (Fig. 3B; *P* ≤ 0.05). Excess cellular cholesterol is esterified to cholesteryl esters that are stored along with triacylglycerols as membrane bound neutral-lipid droplets (LDs) in the cytoplasm (30). Staining of the neutral lipids using lipophilic dyes BODIPY-493/503 and ORO (please see methods for details) revealed increased accumulation of LDs in HBV-miR-3 expressing cells (both Huh7 and HepG2) compared to controls (Fig. 3C (BODIPY-493/503 staining) and supplemental Fig. S3 (ORO staining); *P* ≤ 0.05). Taken together, these results confirm that HBV-miR-3 expression leads to cholesterol accumulation within hepatocytes. Previous reports link increased cholesterol accumulation within hepatocytes to increased cell survival (31, 32). In our microarray data we also found genes related to lipid transfer proteins (LTPs) including Oxysterol-binding protein (*OSBP*), Oxysterol-binding protein-like 2 (*OSBPL2/ORP2*) and *STARD4* to be downregulated (supplemental Table S2.1). LTPs have an established role in the regulation of intracellular cholesterol homeostasis. *OSBP* is reported to be able to both tether organelles and transport lipids between them. *OSBPL2*, localized on LDs, is a sterol receptor required for regulating LD lipolysis; deficiency of *OSBPL2* causes fat accumulation leading to dysregulated energy metabolism (33). Although these genes encoding LTPs do not have HBV-miR-3 binding sites, their downregulation in HBV-miR-3 expressing cells provides evidence of dysregulation of lipid metabolism.

### HBV-miR-3 is a virus-encoded oncomir

**Fig. 4:**
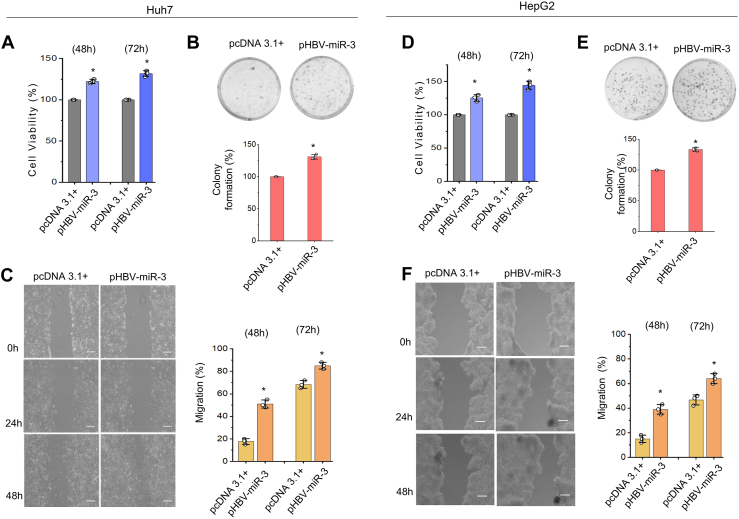
HBV-miR-3 is an oncomir. A: Cell viability (%), (B) Colony forming assay, (C) cell migration assay in Huh7 cells transfected with pHBV-miR-3 and pcDNA3.1+. D: Cell viability (%), (E) Colony forming assay, F: cell migration assay in HepG2 cells transfected with pHBV-miR-3 and pcDNA3.1+. The bar graphs represent mean (±SD) of three independent experiments. ∗*P* ≤ 0.05. The micrographs were captured in bright field at 10x Magnification.

Tumor cells rely on lipid metabolism to meet their increased nutrient demand and to support their uncontrolled growth (34, 35) and an overall intracellular cholesterol retention may contribute to tumor development and progression (36). As our results demonstrate that HBV-miR-3 overexpression leads to decreased expression of *ABCA1*, resulting in the accumulation of intracellular cholesterol, we then explored whether HBV-miR-3 affects cell behaviour. As shown in Figure 4A and 4D, HBV-miR-3 overexpression led to a significant increase in viability of both HepG2 and Huh7 cells (*P* < 0.05). Similarly, we observed a significant increase in reproductive viability (as measured with clonogenic assays; Fig. 4B, E) as well as increased cell migration (Fig. 4E, F) in Huh7 and HepG2 cells expressing HBV-miR-3. Collectively, these data demonstrate that HBV-miR-3 promotes hepatocyte proliferation, colony formation as well as migration. Therefore, HBV-miR-3 may represent a virus-encoded oncogenic miRNA or an oncomir.

### Statins inhibit HBV-miR-3-mediated cellular cholesterol accumulation

**Fig. 5:**
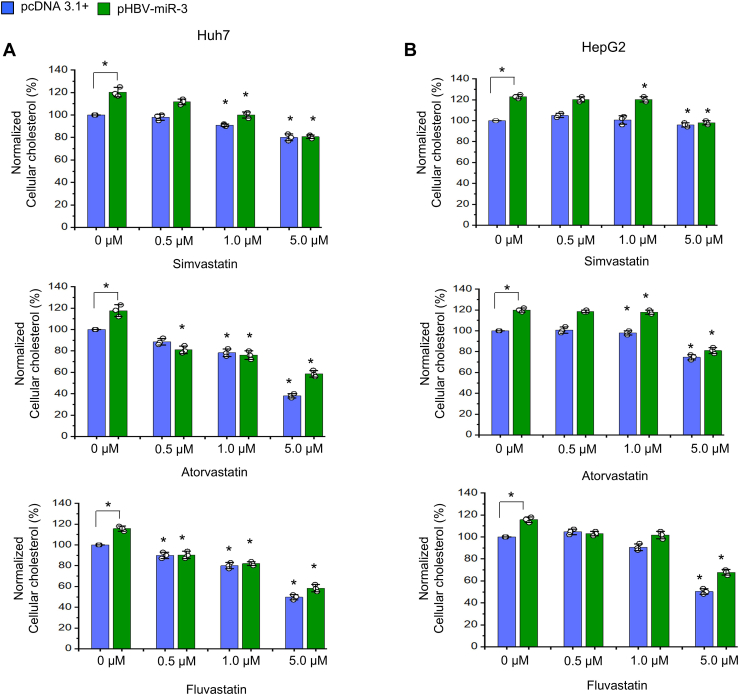
Statins inhibit HBV-miR-3 mediated increase in cellular cholesterol. Cellular cholesterol levels normalised to the cellular protein content in cells: Huh7(A) and HepG2(B) transfected with pHBV-miR-3 and pcDNA3.1+ followed by treatment with the statins including simvastatin, atorvastatin and fluvastatin. Bar graphs represent mean (±SD) of three independent experiments. Here, the statistical significance (*P* values) for decrease in cholesterol levels of the cells treated with “pcDNA3.1+ plus statin” or “pHBV-miR-3 plus statin” at all the three statin doses was analysed with respect to the cells treated with pcDNA3.1+ or pHBV-miR-3 transfected cells (denoted by 0 μM statin) respectively. ∗*P* ≤ 0.05.

Statins are known to inhibit HMG-CoA reductase, the rate-limiting enzyme for de novo cholesterol biosynthesis. Our data suggests that statins can inhibit HBV-miR-3 mediated effects. Since our results suggest that HBV-miR-3 mediates accumulation of intracellular cholesterol which is accompanied by increase in cell proliferation, colony formation and cell migration, we sought to determine if lowering of intracellular cholesterol with the statins alters HBV-miR-3 mediated effects. Huh7 and HepG2 cells transfected with pHBV-miR-3 (or pcDNA3.1+, the vehicle control) were treated with three statins: simvastatin, atorvastatin and fluvastatin at concentrations ranging from 0.5 to 5 μM and were allowed to grow for 48 h, thereafter the cells were subjected to measurement of cholesterol levels as well as cell viability. Our results indicate that all the three statins inhibit HBV-miR-3 induced cholesterol accumulation in both cell lines Huh7 cells and HepG2 cells in a dose-dependent manner. Although, simvastatin was significantly effective at the doses ≥ 1 μM in both cell lines, atorvastatin and fluvastatin significantly suppressed the cholesterol accumulation in Huh7 cells at all the three doses. In HepG2 cells, atorvastatin was effective at doses ≥ 1 μM in inhibiting HBV-miR-3-induced cholesterol accumulation, while fluvastatin was significantly effective at 5 μM (Fig. 5A, B and supplemental Fig. S4). A similar trend in reduction of HBV-miR-3 induced cell viability was observed in both the hepatocyte cell lines after treatment with the three statins (supplemental Fig. S5). Together these results confirm that HBV-miR-3-mediated increase in cell viability is linked with intracellular cholesterol accumulation and statin-mediated lowering of intracellular cholesterol levels supresses the oncogenic effects of this virus encoded miRNA.

### Intrahepatic HBV-miR-3 in liver biopsies from patients with CHBV

**Fig. 6:**
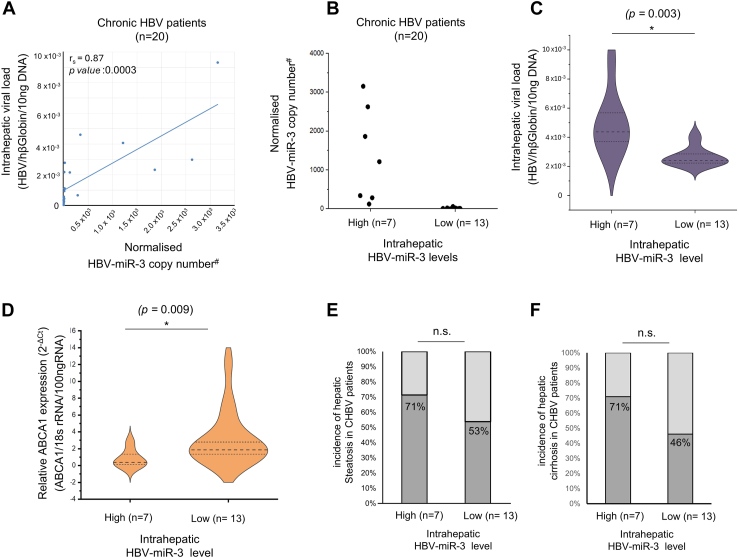
Correlation analysis intrahepatic HBV-miR-3 expression levels in liver biopsies of HBV patients (N = 20) with other parameters. A: Scatter plot demonstrating the correlation (r_s_: Spearman rank correlation coefficient) between intrahepatic HBV-miR-3 levels and intrahepatic HBV DNA (HBV load) in chronic HBV patients (n = 20). B: A plot showing the range of intrahepatic HBV-miR-3 levels in chronic HBV patients, wherein biopsies with HBV-miR-3 copies ≥ 100 copies/100ngRNA are grouped as patients with high HBV-miR-3 and those with HBV-miR-3 copies ≤ 100 copies/100ngRNA (lower than the linear range) are grouped as patients with low HBV-miR-3. (# HBV-miR-3 levels in the biopsies were normalized HBV-miR-3/18srRNA/100 ng RNA). C: Violin plots demonstrating the distribution of intrahepatic viral (HBV) load in biopsies with high HBV-miR-3 expression compared to biopsies with low HBV-miR-3 expression. D: Violin plots showing the distribution of *ABCA1* levels in biopsies with high HBV-miR-3 expression compared to those with low HBV-miR-3 expression. E: Frequency of hepatic steatosis in biopsies with high HBV-miR-3 expression and low HBV-miR-3 expression. F: Frequency of liver cirrhosis in biopsies with high HBV-miR-3 expression and low HBV-miR-3 expression. ∗*P* ≤ 0.05, n.s.: non-significant.

HBV-miR-3 was detected in all the fresh frozen liver biopsies (n = 20) from CHBV patients. HBV-miR-3 levels in liver biopsies demonstrated a positive correlation with intrahepatic HBV DNA levels (r_s_ = 0.87, *P =* 0.0003) (Fig. 6A), suggesting that patients with high HBV loads have high HBV-miR-3 levels in the liver. Seven biopsies showed high HBV-miR-3 expression level (>100 copies/reaction; in the linear range) and 13 biopsies showed low expression of HBV-miR-3 (<100 copies/reaction; below the linear range) (Fig. 6B). The presence of HBV DNA and HBV replication intermediates (pcRNA and pgRNA) was also confirmed in all liver biopsies. The biopsies with high HBV-miR-3 levels (n = 7) had significantly higher intrahepatic HBV loads as compared to those with low HBV-miR-3 levels (Fig. 6C; *P* = 0.003). Furthermore, the biopsies with high HBV-miR-3 levels had significantly lower *ABCA1* transcript levels as compared to those with low HBV-miR-3 (Fig. 6D; *P* = 0.009). Taken together, our findings (from GEO datasets, HBV-miR-3 expressing hepatocyte cell lines and liver biopsies from patients with CHBV) suggest that HBV-miR-3 expression is associated with the suppression of *ABCA1*.

In addition, hepatic steatosis and hepatic cirrhosis were more frequently observed in biopsies from patients with CHBV having high intrahepatic HBV-miR-3 levels compared to those with lower HBV-miR-3 levels (Steatosis: 71% vs. 53%, *P* = 0.37; 71% vs. 46%, *P* = 0.27) (Fig. 6E, F); however, this was not statistically significant. Perhaps, a largescale study would provide a more comprehensive view of the associations, if any, between hepatic HBV-miR-3 levels and hepatic steatosis/cirrhosis.

## Discussion

Over the past two decades, numerous virus-encoded miRNAs have been identified however their mechanistic involvement in viral pathogenesis is only beginning to be explored (37, 38, 39) HBV-miR-3, a miRNA encoded by HBV was identified through deep sequencing of HBV-HCC tissues (3). We found that the HBV-miR-3 seed site which is the major determinant in miRNA-target mRNA interaction is highly conserved (>97.7%) within and across HBV genotypes, suggesting a potential biological role for this HBV encoded miRNA. Our transcriptome analysis of hepatocytes ectopically expressing HBV-miR-3 identified differentially expressed genes linked to lipid transport/metabolism. *ABCA1* was shortlisted for functional studies as (a) *ABCA1* transcript levels were significantly down-regulated in our microarray data from hepatocytes expressing HBV-miR-3; (b) *ABCA1* was identified as a host gene harboring potential HBV-miR-3 binding sites using two algorithms; and (c) Analysis of publicly available datasets (n = 3 datasets), indicates that *ABCA1* transcript levels were significantly lower in liver biopsies from HBV-infected cases (compared to uninfected controls) and in HBV-related HCC (as compared with the paired non-tumor liver samples).

*ABCA1* is an important regulator of hepatocyte cholesterol efflux. We validate *ABCA1* as bonafide target of HBV-miR-3 by demonstrating the binding of HBV-miR-3 to the 3′ UTR of *ABCA1* using luciferase reporter assays. Furthermore, HBV-miR-3 expression in Huh7 and HepG2 cells led to (a) significant reduction in *ABCA1* levels and (b) significant increase in the accumulation of intracellular cholesterol and lipid droplets. These findings suggest that HBV-miR-3 leads to accumulation of cholesterol and lipid droplets in hepatocytes by targeting *ABCA1*.

HBV infection has been linked to dysregulation of lipid metabolism and increase in hepatic cholesterol deposition (40, 41). Abnormal lipid metabolism in the liver is associated with the development of non-alcoholic fatty liver disease (NAFLD), which is increasingly being recognized as the major risk factor for development of HCC. HBV-steatosis co-occurrence has been frequently observed in clinical practice. However, whether HBV-related hepatocarcinogenesis precedes hepatic steatosis or hepatic steatosis increases the risk of HCC in patients with chronic HBV infection is incompletely understood (42, 43). Nevertheless, it is widely accepted that metabolic risk factors including hepatic steatosis offer contributory environment for pathogenic liver condition culminating into HCC (44). While the link between chronic HBV infection and dysregulation of lipid metabolism, hepatic steatosis and HBV-related HCC is well-documented, the underlying mechanisms remain elusive. Our findings suggest that HBV-encoded HBV-miR-3 may play a key mechanistic role in HBV-mediated dysregulation of lipid metabolism in infected hepatocytes.

We then analyzed the impact of HBV-miR-3 expression on cell behavior. Interestingly, HBV-miR-3 expression increases cell proliferation, colony formation and cell migration in both Huh7 and HepG2 cells. The link between cholesterol levels and various cancers including HCC is well documented (45, 46). Cholesterol is a critical component of the cell membranes and contributes to translocation of other lipids. The dysregulation of cholesterol levels may lead to metabolic reprogramming, affect immune microenvironment and impact the homeostasis of gut microbiome, thus contributing to HCC (45).

We then sought to understand if HBV-miR-3 induced cholesterol accumulation is the primary cause of increased proliferation. This is particularly relevant as there could be other unidentified HBV-miR-3 targets unrelated to cholesterol metabolism that have a tumor suppressor role. For this purpose, we used three statins: simvastatin, atorvastatin and fluvastatin. The statins are clinically prescribed cholesterol lowering drugs that reduces de novo cholesterol biosynthesis. The statins are categorized as naturally derived statins (e.g. simvastatin), semisynthetic derivatives, and synthetically derived statins (fluvastatin, pitavastatin, rosuvastatin, and atorvastatin). Although all statins contain a HMG-CoA-like moiety responsible for inhibiting HMGR, the synthetic statins differ from the natural statins in their structural composition (47). The addition of all three statins (simvastatin, atorvastatin and fluvastatin) to Huh7 and HepG2 cells led to the suppression of HBV-miR-3-induced increase in cellular cholesterol as well as cell proliferation. This finding suggests that (a) the HBV-miR-3-induced increased in cell proliferation is primarily linked to *ABCA1* targeting and the resultant intracellular accumulation of cholesterol and (b) Statin-meditated decrease in intracellular cholesterol levels inhibit HBV-miR-3-induced increased in hepatocyte proliferation. Previous reports suggest that statins may reduce HCC including HBV-related HCC (48, 49). The potential anticancer effects of statins are being increasingly recognized (50), although the specific mechanisms are not well understood.

To further ascertain the clinical relevance of HBV-miR-3, we analyzed 20 liver biopsies from CHBV patients. HBV-miR-3 was detected in all the 20 liver biopsies tested. Further, high HBV-miR-3 levels in the liver were associated with high intrahepatic HBV DNA levels; thus, intrahepatic HBV loads may serve as surrogates for high HBV-miR-3 levels in the liver. Furthermore, patients with high HBV-miR-3 levels in liver had significantly lower levels of *ABCA1* transcripts, corroborating our findings in hepatocyte cell cultures. Among CHBV patients, a non-significant trend towards higher frequency of hepatic steatosis and hepatic cirrhosis was observed in patients with high HBV-miR-3 levels compared to those with lower HBV-miR-3 levels. A large-scale study might provide a more comprehensive picture of the association, if any, between hepatic HBV-miR-3 levels and hepatic steatosis/cirrhosis.

In summary, we identify *ABCA1* as a bonafide target of HBV-miR-3. Our results support the notion that HBV-miR-3-mediated targeting of *ABCA1* results in increased accumulation of cholesterol and lipid droplets in hepatocytes leading to increased cell proliferation and colony formation. Statin-mediated reduction in intracellular cholesterol levels inhibits HBV-miR-3-mediated increased proliferation. Our findings on liver biopsies from CHBV patients suggests that high HBV-miR-3 expression in the liver is linked to high intrahepatic HBV DNA levels and lower *ABCA1* expression in the liver. HBV-miR-3 may represent the missing link between CHBV infection and dysregulation of lipid metabolism in hepatocytes, acting as a key etiological factor that contributes to complex underlying mechanisms in the development of HBV-mediated liver disease. Taken together, this work highlights how a virus-encoded miRNA alters lipid metabolism and cell behaviors. Importantly, the ability of statins to inhibit HBV-miR-3-induced increased proliferation merits further studies on the potential benefits of statins in reducing the risk of HBV-related HCC. In addition to deciphering an oncogenic role for HBV-miR-3 through dysregulation of lipid metabolism, our findings on how widely used statins can inhibit HBV-miR-3-mediated effect highlight the clinical relevance of this work. This work lays the foundation for further studies at the intersection of infectious diseases, metabolism and cancer.

## Data availability

All data reported in this paper will be shared by the lead contact upon request.

## Supplemental data

This article contains supplemental data. The references cited in the supplemental Table S1 (containing the list of primers) include (3, 51, 52, 53) from the reference list.

## Conflict of interest

The authors declare that they do not have any conflicts of interest with the content of this article.

## Figures and Tables

**Figure:**
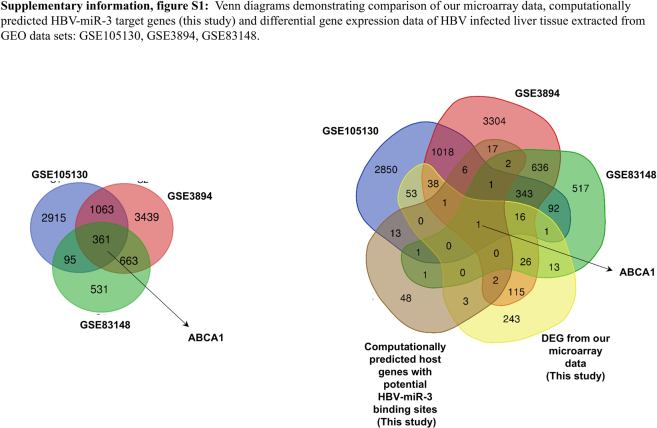
Supplemental Fig. 1

**Figure:**
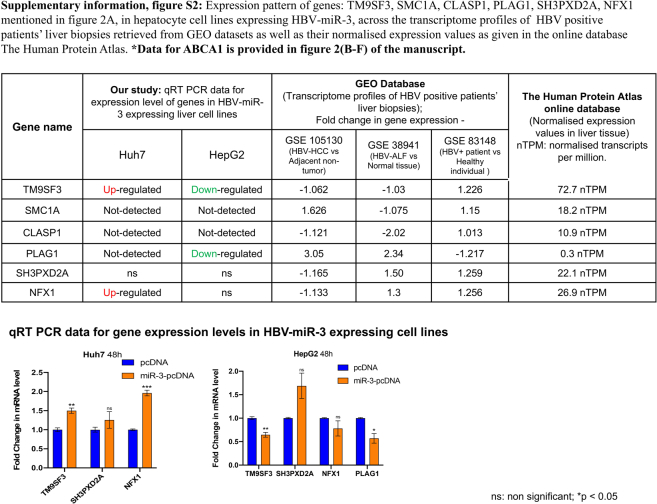
Supplemental Fig. 2

**Figure:**
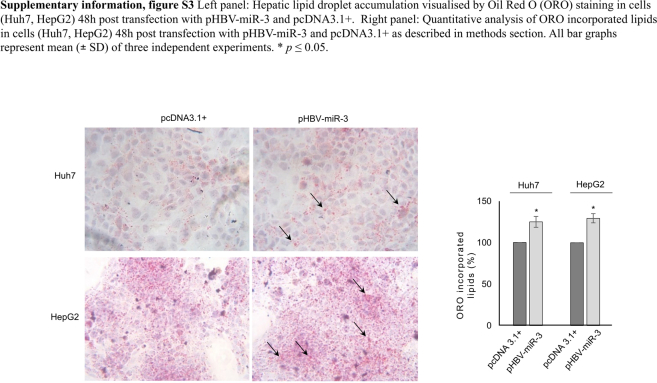
Supplemental Fig. 3

**Figure:**
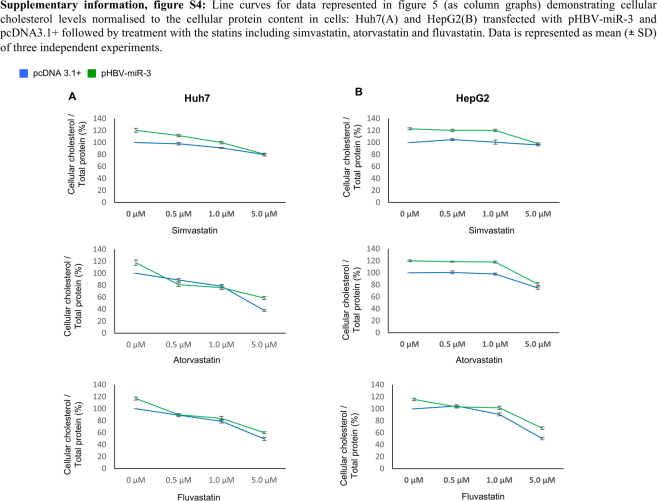
Supplemental Fig. 4

**Figure:**
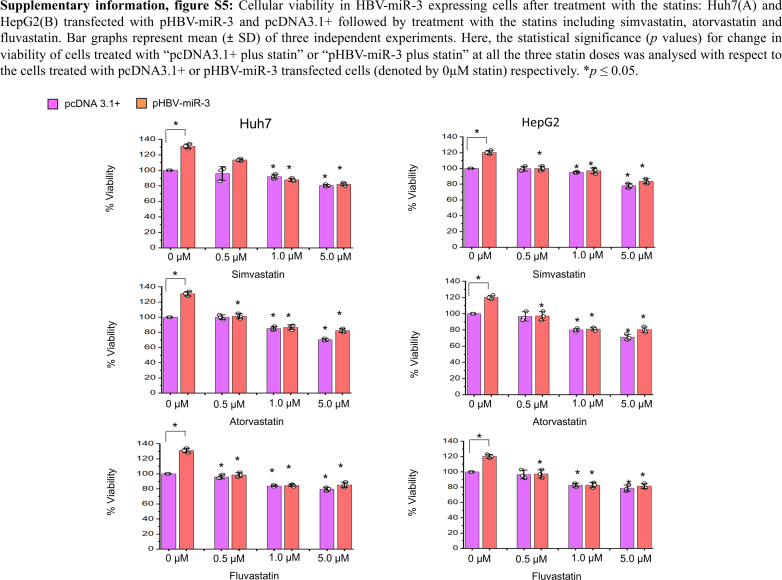
Supplemental Fig. 5

**Figure:**
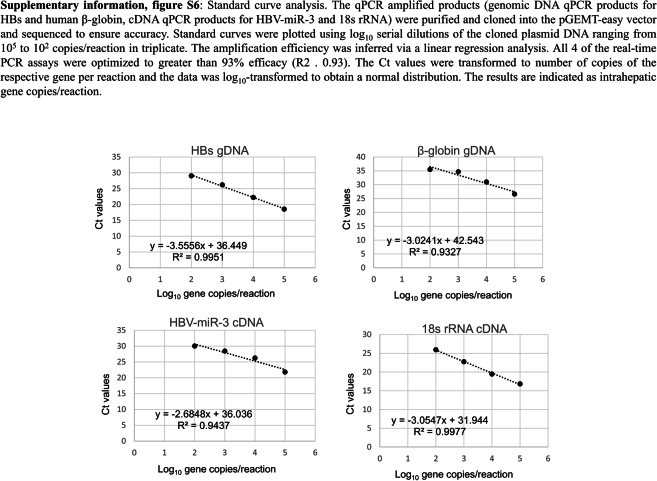
Supplemental Fig. 6
