# Supplementary material for: HBV-miR-3 induces hepatic cholesterol accumulation by targeting ABCA1: Evidence for potential benefits of statin usage
Source: J Lipid Res. 2025 Jul 21;66(8):100866. doi: 10.1016/j.jlr.2025.100866 (PMC12391803; doi:10.1016/j.jlr.2025.100866)
Supplement: Supplemental Material [file mmc2.docx]

**HBV-miR-3 induces hepatic cholesterol accumulation by targeting *ABCA1*: evidence for potential benefits of statin usage**

Shruti Chowdhari^a^, Auroni Deep^a^, Jasmine Samal^b^, Ekta Gupta^b^, Perumal Vivekanandan^a^#

**^a^**Kusuma School of Biological Sciences, Indian Institute of Technology Delhi, New Delhi, India

**^b^** Institute of Liver and Biliary Sciences (ILBS) repository, New Delhi, India

**SUPPORTING INFORMATION**

**Supplementary Table 1: List of primers**

|  | **Name of primer** | **sequence (5’ to 3’)** | **Reference** |
| --- | --- | --- | --- |
| 1 | HBV-miR-3  cDNA specific primer | GTCGTATCCAGTGCAGGGTCCGAGG  TGCACTGGATACGACAAACGCCG | (3) |
| 2 | HBV-miR-3 FP | TGCGGCTGGATGTGTCTGCG |  |
| 3 | HBV-miR-3 RP | CCAGTGCAGGGTCCGAGGT |  |
| 4 | 18s rRNA FP | GTAACCCGTTGAACCCCATT | (51) |
| 5 | 18s rRNA RP | CCATCCAATCGGTAGTAGCG |  |
| 6 | ABCA1 qFP | CAGAGGTGGCTCTGATGACC | This study |
| 7 | ABCA1 qRP | TGTTTTGCTTTGCTGACCCG | This study |
| 8 | ABCA1-Luc-WT FP | GCGATCGCCATGGCCAGCGATTATTGAT | This study |
| 9 | ABCA1-Luc-WT RP | TACTCGAGGCATGTTACTGCCACCAGAA | This study |
| 10 | ABCA1-Luc-Mut FP | CTGGTTTCCCGGTGACTTGCTGGCAATGAGTG | This study |
| 11 | ABCA1-Luc-Mut RP | CACTCATTGCCAGCAAGTCACCGGGAAACCAG | This study |
| 12 | B Globin- FP | GGTTGGCCAATCTACTCCCAGG | (52) |
| 13 | B Globin-RP | TGGTCTCCTTAAACCTGTCTTG |  |
| 14 | HBV pre genomic RNA FP | CACCTCTGCCTAATCATC | (53) |
| 15 | HBV pre core RNA FP | GGTCTGCGCACCAGCACC |  |
| 16 | HBV RNA RP | GGAAAGAAGTCAGAAGGCAA |  |

**Supplementary Table 2**

|  | **GO: Biological processes** | ***p* value** | **No of genes** |
| --- | --- | --- | --- |
| 1 | positive regulation of transcription by RNA polymerase II | 6.89E-04 | 75 |
| 2 | **lipid metabolic process** | 1.79E-04 | 58 |
| 3 | positive regulation of cell population proliferation | 6.83E-04 | 41 |
| 4 | Angiogenesis | 5.57E-04 | 24 |
| 5 | metabolic process | 2.45E-04 | 20 |
| 6 | integrin-mediated signaling pathway | 2.27E-04 | 14 |
| 7 | cerebral cortex development | 4.96E-05 | 12 |
| 8 | insulin receptor signaling pathway | 4.56E-05 | 11 |
| 9 | negative regulation of ERK1 and ERK2 cascade | 5.93E-04 | 11 |
| 10 | ubiquitin-dependent ERAD pathway | 6.62E-04 | 11 |
| 11 | **intermembrane lipid transfer** | 2.94E-04 | 9 |
| 12 | **intracellular cholesterol transport** | 1.57E-04 | 5 |
| 13 | cellular response to chemokine | 4.76E-04 | 5 |
| 14 | cellular hypotonic response | 3.94E-04 | 4 |
| 15 | positive regulation of potassium ion transport | 6.34E-04 | 4 |
| 16 | regulation of lamellipodium assembly | 6.34E-04 | 4 |
| 17 | chemokine (C-X-C motif) ligand 12 signalling pathway | 8.51E-05 | 3 |
| 18 | alanine transport | 7.96E-04 | 3 |
| 19 | positive regulation of basement membrane assembly involved in embryonic body morphogenesis | 7.96E-04 | 3 |
| 20 | protein localization to site of double-strand break | 7.96E-04 | 3 |

**Supplementary Table 2.1**

| **GO Biological process: Intracellular cholesterol transport** | | |
| --- | --- | --- |
| **Gene** | **Description** | **Microarray data** |
| *ABCA1* | ATP binding cassette subfamily A member 1 | -1.36 |
| *ABCG1* | ATP binding cassette subfamily G member 1 | 1.36 |
| *OSBPL2* | oxysterol binding protein like 2 | -1.26 |
| *OSBP* | oxysterol binding protein | -1.31 |
| *STARD4* | StAR related lipid transfer domain containing 4 | -1.28 |
| **GO Biological process: Intermembrane lipid transfer** | | |
| **Gene** | **Description** | **Microarray data** |
| *ABCG1* | ATP binding cassette subfamily G member 1 | 1.36 |
| *TTPA* | Alpha tocopherol transfer protein | 1.28 |
| *OSBPL2* | Oxysterol binding protein like 2 | -1.26 |
| *ATG2B* | Autophagy related 2B | -1.35 |
| *OSBP* | Oxysterol binding protein | -1.31 |
| *GRAMD1C* | GRAM domain containing 1C | 1.26 |
| *STARD4* | StAR related lipid transfer domain containing 4 | -1.28 |
| *ABCA1* | ATP binding cassette subfamily A member 1 | -1.36 |
| *MTTP* | Microsomal triglyceride transfer protein | 1.28 |
| **GO Biological process: Lipid metabolic process** | | |
| **Gene** | **Description** | **Microarray data** |
| *LBR* | Lamin B receptor | -1.36 |
| *PLA2G4C* | Phospholipase A2 group IVC | 1.26 |
| *SYNJ1* | Synaptojanin 1 | -1.28 |
| *CYP4F12* | Cytochrome P450 family 4 subfamily F member 12 | -1.26 |
| *CYP7B1* | Cytochrome P450 family 7 subfamily B member 1 | -1.3 |
| *FA2H* | Fatty acid 2-hydroxylase | 1.29 |
| *FUCA1* | Alpha-L-fucosidase 1 | -1.41 |
| *NUS1* | NUS1 dehydrodolichyl diphosphate synthase subunit | 1.39 |
| *LIPA* | Lipase A, lysosomal acid type | -1.31 |
| *ABHD16A* | Abhydrolase domain containing 16A, phospholipase | -1.35 |
| *MOGAT3* | Monoacylglycerol O-acyltransferase 3 | -1.35 |
| *TPP1* | Tripeptidyl peptidase 1 | -1.29 |
| *ABHD4* | Abhydrolase domain containing 4, N-acyl phospholipase B | -1.88 |
| *RETSAT* | Retinol saturase | -1.35 |
| *AGPS* | Alkylglycerone phosphate synthase | -1.27 |
| *B4GALT5* | Beta-1,4-galactosyltransferase 5 | -1.3 |
| *FAM213B* | Peroxiredoxin like 2B (PRXL2B) | 1.35 |
| *SACM1L* | SAC1 like phosphatidylinositide phosphatase | -1.34 |
| *PLPP1* | Phospholipid phosphatase 1 | -1.31 |
| *TTPA* | Alpha tocopherol transfer protein | 1.26 |
| *PLCXD3* | Phosphatidylinositol specific phospholipase C X domain containing 3 | -1.63 |
| *PC* | Pyruvate carboxylase | -1.55 |
| *CYP3A5* | Cytochrome P450 family 3 subfamily A member 5 | -1.93 |
| *LCLAT1* | Lysocardiolipin acyltransferase 1 | -1.31 |
| *B3GNT5* | UDP-GlcNAc:betaGal beta-1,3-N-acetylglucosaminyltransferase 5 | 1.34 |
| *LRAT* | Lecithin retinol acyltransferase | -1.29 |
| *NCEH1* | Neutral cholesterol ester hydrolase 1 | -1.56 |
| *HPGD* | 15-hydroxyprostaglandin dehydrogenase | -1.38 |
| *ACSS2* | Acyl-CoA synthetase short chain family member 2 | -1.37 |
| *AKR1B15* | Aldo-keto reductase family 1 member B15 | -1.36 |
| *INSIG2* | Insulin induced gene 2 | -1.45 |
| *AKR1B10* | Aldo-keto reductase family 1 member B10 | -1.43 |
| *UGT2B10* | UDP glucuronosyltransferase family 2 member B10 | 1.37 |
| *LSS* | Lanosterol synthase | -1.29 |
| *ERLIN2* | ER lipid raft associated 2 | -1.4 |
| *FADS2* | Fatty acid desaturase 2 | -1.3 |
| *PLPP5* | Phospholipid phosphatase 5 | 1.3 |
| *GALC* | Galactosylceramidase | -1.3 |
| *PTPMT1* | Protein tyrosine phosphatase mitochondrial 1 | 1.28 |
| *AKR1C2* | Aldo-keto reductase family 1 member C2 | -1.42 |
| *PLCXD1* | Phosphatidylinositol specific phospholipase C X domain containing 1 | -1.38 |
| *PAFAH1B2* | Platelet activating factor acetylhydrolase 1b catalytic subunit 2 | 1.31 |
| *GPAM* | Glycerol-3-phosphate acyltransferase, mitochondrial | 1.43 |
| *PLCXD2* | Phosphatidylinositol specific phospholipase C X domain containing 2 | 1.31 |
| *CYP1A1* | Cytochrome P450 family 1 subfamily A member 1 | -1.34 |
| *GPX5* | Glutathione peroxidase 5 | -1.26 |
| *AGK* | Acylglycerol kinase | -1.34 |
| *PIK3C2A* | Phosphatidylinositol-4-phosphate 3-kinase catalytic subunit type 2 alpha | -1.4 |
| *MTMR3* | Myotubularin related protein 3 | -1.31 |
| *CERS2* | Ceramide synthase 2 | -1.4 |
| *HSD3B1* | Hydroxy-delta-5-steroid dehydrogenase, 3 beta- and steroid delta-isomerase 1 | 1.28 |
| *PLA1A* | Phospholipase A1 member A | 1.34 |
| *UGT8* | UDP glycosyltransferase 8 | 1.62 |
| *CYP3A7* | cytochrome P450, family 3, subfamily A, polypeptide 7 | -1.39 |
| *ABCA1* | ATP binding cassette subfamily A member 1 | -1.36 |
| *MTTP* | Microsomal triglyceride transfer protein | 1.28 |
| *AKR1B1* | Aldo-keto reductase family 1 member B | -1.94 |
| *PIK3CB* | Phosphatidylinositol-4,5-bisphosphate 3-kinase catalytic subunit beta | -1.28 |
