# Supplementary material for: HBV-miR-3 induces hepatic cholesterol accumulation by targeting ABCA1: Evidence for potential benefits of statin usage
Source: J Lipid Res. 2025 Jul 21;66(8):100866. doi: 10.1016/j.jlr.2025.100866 (PMC12391803; doi:10.1016/j.jlr.2025.100866)
Supplement: Supplemental Data [file mmc1.pdf]

INSTITUTE OF LIVER & BILIARY SCIENCES
(Deemed-to-be-University)
(An Autonomous Society under Government of NCT of Delhi)
D-1, Vasant Kunj, New Delhi, India Phone No: 46300000
Email: iec.ilbs@gmail.com, ethics@ilbs.in, Website: http://www.ilbs.in

ILBS-ACAD012/8/2023-ACAD/E-12745/39 **Dated: 14-02-2023**

Communication of Decision of the Institutional Ethics Committee (IEC) / Institutional Review Board (IRB)

IEC/2023/98/MA09

**Protocol Title:-** "To study the role of HBV-encoded miRNAs in hepatocellularcarcinoma (HCC)".
**Student Investigator-** Dr. Jasmine Samal, Senior Fellow (Clinical Virology)<br>**Faculty Supervisor-** Dr. Ekta Gupta, Professor (Clinical Virology)
**Name & Address of Institution**: INSTITUTE OF LIVER AND BILIARY SCIENCES, D-1, VASANT KUNJ, NEW DELHI
☑ New review ☐ Revised review ☐ Expedited review
Date of review (DD/MM/YYYY): **24-01-2023**<br>Date of previous review, if revised application: **NA**
**Decision of the IEC/ IRB:**<br>☑ Recommended ☐ Recommended with modifications<br>☐ Revision to be submitted again ☐ Rejected
**Suggestions/ Reasons/ Remarks:** (**Approved)**
**Remarks: The members who were present and participated in the voting are as follows:**

1.	Dr. A. K. Agarwal	Chairman	6.	Dr. Ashok Choudhury	Member
2.	Dr. Y. K. Gupta	Member	7.	Sh. Sanjay Poddar	Member
3.	Dr. Veena Malhotra	Member	8.	Ms. Shiv Rani	Member
4.	Dr. Vijay Kumar	Member	9.	Sh. Koldie Ghai	Member
5.	Dr. Sanjeev Sachdeva	Member	10.	Dr. Rajeev Khanna	Member Secretary

Please note *

1. Inform IEC/IRB immediately in case of any adverse events and serious adverse events.
2. Inform IEC/IRB in case of any change of research team, study procedure/ protocol/ site/ discontinuation of the study.
3. Provide a 'final report' when the project is completed.

Rajeev

(Dr. Rajeev Khanna)
Member Secretary, IEC/ IRB ILBS

Dr. RAJEEV KHANNA
Member Secretary
Institutional Ethics Committee
Institute of Liver & Biliary Sciences
Sector D-1, Vasant Kunj
New Delhi-110 070

*****
